# Leveraging High-Frequency Postoperative ICU Arterial Blood Pressure Data from Electronic Health Records for Research: A Brief Report

**DOI:** 10.1007/s10916-026-02472-4

**Published:** 2026-10-14

**Authors:** Gen Li, Robert E. Freundlich, Benjamin French, David R. McIlroy

**Affiliations:** 1 Department of Anesthesiology, Vanderbilt University Medical Center, 1211 21 st Avenue South, Medical Arts Building, Suite 731F, Nashville, TN 37212 USA; 2 Department of Anesthesiology, Department of Biomedical Informatics, Vanderbilt University Medical Center, Nashville, TN USA; 3 Department of Biostatistics, Vanderbilt University Medical Center, Nashville, TN USA

**Keywords:** Postoperative blood pressure, High-frequency data, Multiple imputation, Reusable workflow

## Abstract

Postoperative blood pressure fluctuations after cardiac surgery are highly clinically relevant, yet analyzing high-frequency arterial pressure data remains challenging. In this study, we introduce a novel methodology for processing minute-by-minute postoperative intensive care unit (ICU) arterial pressure data. The pipeline implements a series of systematic data-cleaning steps to resolve artifacts and ensure physiological plausibility, and deploys a multi-stage imputation framework to address missing intervals. Our approach constructs longitudinal blood pressure profiles that quantify the time spent within predefined blood pressure ranges, minimizing assumptions and enhancing data utility for research. We detail a rigorous and reusable workflow for data extraction, cleaning, and imputation, which may address limitations of previous methods and offer a framework to build a robust exposure metric for future research utilizing postoperative arterial pressure data.

**Supplementary Information:**

The online version contains supplementary material available at https://doi.org/10.1007/s10916-026-02472-4.

## Background

The literature has consistently demonstrated an association between perioperative mean arterial pressure (MAP) and postoperative adverse events [1, 6]. Investigating these associations requires leveraging large-scale data derived from clinical monitors. However, the core challenge for researchers is how best to apply robust methodologies to extract, preprocess, and analyze these high-frequency data.

We evaluated how existing studies handle blood pressure data by reviewing common methods applied by researchers across various clinical settings, as illustrated in Table 1. These publications cover cardiac and non-cardiac surgeries, invasive arterial lines and non-invasive tracking, and cohorts utilizing intraoperative or postoperative data [3, 8]. Specifically, we examined their data extraction protocols, artifact-cleaning algorithms, and missing data strategies, as well as how they transform raw measurements into final exposure metrics. This evaluation highlights that conventional pipelines rely on rigid data-processing shortcuts, including Last Observation Carried Forward (LOCF) and linear geometric interpolation, which overlook critical information by smoothing out blood pressure fluctuations. Additionally, these commonly used exposure metrics, such as time-weighted averages, time spent below arbitrary thresholds, or absolute 24-h nadirs, reflect underlying assumptions that are likely incorrect and prone to bias.

**Table 1:** Characteristics and Key Analytical Approaches of Perioperative Blood Pressure Data Extraction and Processing across Research Studies

Authors, Year	Key Data Extraction & Processing Approaches	Advantages	Limitations
Walsh et al.,2013 [3]	• Uses mixed resolution: 1–2 min arterial line sampling; 2–5 min intervals for non-invasive cuffs • Employs manual provider-driven validation to flag and exclude raw mechanical artifacts • Applies Last Observation Carried Forward (LOCF) patching to construct continuous grids; sums per-minute placeholders below thresholds to compute cumulative duration	• Offers high automatability across multi-center studies • Yields continuous traces for simple duration tallies	• Extends historical entries over dynamic drops via LOCF, flattening continuous vital sign variance • Assumes monotonic threshold-outcome risks, masking the absolute severity and depth of hypotension
Sun et al., 2015 [4]	• Uses high-resolution: 1-min automated sampling from continuous invasive arterial lines • Deploys automated algorithm filtering: deletes isolated MAP values fluctuating > 50% from adjacent minute markers • Executes targeted sequence deletion: leaves dropouts or absent intervals ≤ 2 min left blank and unreplaced	• Maximizes measurement precision • Eliminates confounding and mechanical errors inherent to non-invasive cuffs	• Relies on binary, absolute cutoffs, which flattens physiological variations • Biases results conservatively toward the null by aggressively stripping data gaps
van Waes et al., 2016 [5]	• Uses mixed resolution: 1-min arterial line sampling; 3–5 min intervals for non-invasive cuffs • Deploys artifact range filtering: deletes isolated 1-min drops to eliminate line-flush noise • Imputes preceding non-artifact values; integrates cumulative durations into multi-tiered Area Under the Curve (AUC) sensitivity indices	• Reduces signal noise • Isolates absolute drops from preinduction baselines	• Ignores the impact of rapid blood pressure variability due to binned categories assuming stepwise monotonic risk • Flattens dynamic recovery patterns through carry-forward imputation across wide cuff intervals
Salmasi et al. 2017 [6]	• Uses mixed resolution: 1-min arterial line sampling; 1–5 min intervals for non-invasive cuffs • Applies rule-based automated filters; excludes extreme artifacts and SBP jumps ≥ 80 mmHg/min • Utilizes linear interpolation across gaps; enforces list-wise deletion for continuous missing data exceeding 10 min	• Constructs continuous timelines optimized for population-level normalized Time-Weighted Average (TWA) regression	• Forces discretization bias by categorizing continuous TWA into rigid quartile bins • Hides brief, transient intra-interval blood pressure fluctuations via geometric slope across missing intervals
Jin et al. 2019 [7]	• Uses intermittent resolution: logs postoperative electronic tracks at static 15-min intervals • Deploys algorithmic range screening to purge erroneous readings (SBP > 299 or < 25 mmHg) • Enforces extrema boundary capture by rejecting time-series interpolation and extracting single static absolute upper/lower limits from discrete recordings	• Requires zero background processing or time-series reconstruction infrastructure • Prevents the introduction of artificial values into the data	• Fails to capture short-term tracking instability due to reliance on intermittent observation logs • Treats brief dips identically to prolonged hypotensive episodes by extracting isolated single-point nadirs
Vandenberghe et al. 2022 [8]	• Uses intermittent resolution: logs records at static 1-h intervals within an electronic ICU registry • Utilizes bedside nurse validation; performs manual cross-checks every hour to filter transient mechanical noise • Enforces phase-segmented zero-imputation; leaves missing data blocks unreplaced and blank	• Isolates structural noise through nurse validation • Structures data into independent blocks to prevent missing data from skewing initial baseline summaries	• Misses acute, minute-level continuous blood pressure fluctuations due to the use of static 1-h logs • Disrupts trajectory modeling by leaving gaps unreplaced; fails to capture cumulative exposure severity by bypassing duration/AUC

To address these gaps, advanced methodologies are required to process blood pressure data at higher temporal resolution, paired with metrics that minimize assumptions about the variability of blood pressure exposure. This report introduces and evaluates a reusable framework for the extraction, cleaning, imputation, and mapping of high-frequency postoperative intensive care unit (ICU) MAP data into seven mutually exclusive blood pressure bands. By leveraging high-frequency data, this reusable workflow generates granular, research-ready longitudinal profiles of postoperative MAP exposure to minimize processing bias.

## Methods

### Study Setting and Data Sources

For a recent institutional study investigating the association between postoperative blood pressure management and adverse outcomes in patients undergoing cardiac surgery, we developed a standardized workflow to handle minute-by-minute ICU MAP data [9]. Cardiac surgery patients are unique in that continuous and invasive blood pressure monitoring routinely continues for a variable period of time postoperatively in the ICU. [10] The intensive monitoring and frequent data capture provide an opportunity to evaluate hemodynamic patterns using high-resolution MAP data from the electronic health record (EHR).

The ArchiveVital database, hosted by Vanderbilt University Medical Center (VUMC) Health IT, captures high-frequency data directly from bedside monitors. For this study, minute-by-minute MAP values during the first 24 h in the ICU were extracted from the ArchiveVital database. Additional clinical variables were obtained from Clarity (*Epic Systems, Verona, WI*) and our internally developed ICU DataMart, which automatically and routinely aggregates EHR data for research [11]. Despite direct capture, missing or inaccurate data occur due to arterial line removal or dysfunction, discordant values from multiple coexisting arterial lines, temporary absence from the ICU for additional diagnostic or therapeutic procedures, early discharge from the ICU, or other technical failures in the transfer or storage of valid data. To address these challenges, we implemented a systematic data preprocessing workflow to ensure the reliability and integrity of the MAP data.

### Preliminary Data Extraction and Validation

Data were extracted from the VUMC Perioperative Data Warehouse (*Nashville, TN*) using Structured Query Language. We iteratively refined the algorithms based on sample data validation before applying the finalized, optimized versions to the entire cohort.

In most EHR systems, similar data may be stored under different variable names across multiple tables. For this study, we primarily used continuous invasive MAP data from two flowsheet rows: arterial line (ARTm) and arterial blood pressure (ABPm), both reflecting real-time bedside monitoring measurements. These high-frequency monitoring data were supplemented with manually charted nursing entries recorded at discrete intervals. Integrating high-frequency monitoring data with less granular manual charting required addressing discrepancies between sources in values and timing; therefore, we performed rigorous validation and a series of data-cleaning steps to resolve artifacts and inconsistencies, ensuring the data aligned with physiological plausibility.

For each patient, postoperative MAP collection began at the later of ICU admission or anesthesia stop time, accounting for those who may have been cared for in the ICU prior to surgery. The endpoint was the earlier of 24 h after collection started or ICU discharge, as some patients may have been discharged within 24 h postoperatively. After raw data collection, ARTm and ABPm values were processed separately. Within each source, spurious or implausible MAP values (< 30 or > 150 mmHg) were removed. If multiple values occurred within one minute from the same source, the median value was assigned for that minute. Moreover, for any rolling 15-min period with no MAP variation, values were retained if only one was recorded; otherwise, multiple identical values were deemed a true lack of variability and set to missing. Furthermore, outliers, defined as isolated values deviating > 50% from both the preceding and subsequent minutes, were identified as artifacts and therefore excluded. When both ARTm and ABPm values occurred at the same timestamp, the higher value was retained, reflecting clinical practice and real-world decision-making.

Finally, lower-frequency nursing flowsheets data were used to fill gaps where neither ARTm nor ABPm data were available.

### Multiple Imputation of Missing Data

Following initial cleaning, each patient had up to 1,440 min of postoperative MAP data, including missing values. Key perioperative covariates (Supplemental Digital Content 1) were selected a priori based on clinical relevance and established literature for imputation. These included both patient-level static variables, such as demographics, clinical characteristics, and medications, as well as time-dependent variables, notably minute-by-minute intraoperative and postoperative MAP measurements. Integrating this extensive set captured patient heterogeneity to inform the missingness remediation. Of note, the variables in our imputation model were selected specifically for this study. Future applications should tailor variable selection to their intended analyses and perform sensitivity analyses if outcome variables are included.

Given the hierarchical structure of the data, we implemented a two-step imputation protocol to address missing values [12]. First, multiple imputation (MI) via chained equations was applied to the patient-level static variables to generate five imputed datasets [13]. Second, we applied an imputation method combining the expectation–maximization algorithm with bootstrapping, to leverage these complete static variables to impute the time-dependent longitudinal variables within each dataset [14]. This iterative process leveraged observed relationships across patients to fill in missing values in the time series. Each dataset generated in the first step was further imputed ten times, resulting in 50 fully imputed datasets for final analysis. Our two-step approach preserved both temporal dynamics and patient-specific characteristics, maintaining physiological patterns while fully representing imputation uncertainty. The multiple imputation was conducted using R v4.3.3 (mice and Amelia packages) on Databricks Runtime 14.3 LTS (Apache Spark 3.5.0).

### Construction of Longitudinal Postoperative MAP Profiles

After imputation, each patient had a complete minute-by-minute MAP profile for the first 24 h in the ICU. We defined seven mutually exclusive MAP range bands: < 55, 55–59, 60–64, 65–69, 70–74, 75–79, and ≥ 80 mmHg. The primary exposure metric was the total minutes each patient spent within each MAP band. Similarly, we characterized a secondary longitudinal profile based on the percentage change in minute-by-minute values from each patient’s preoperative baseline MAP, defined as the last recorded value before entering the operating room. This was also categorized into seven mutually exclusive bands: > −50%, > −25% to −50%, > −10% to −25%, −10% to + 10%, > 10% to + 25%, > 25% to + 50%, and > + 50%.

To assess the impact of imputed data, we evaluated the between-imputation and within-imputation variances, the relative increase in variance, and the fraction of missing information as diagnostic measures of imputation influence on the finalized blood pressure metrics across the 50 datasets.

## Results

**Fig. 1:**
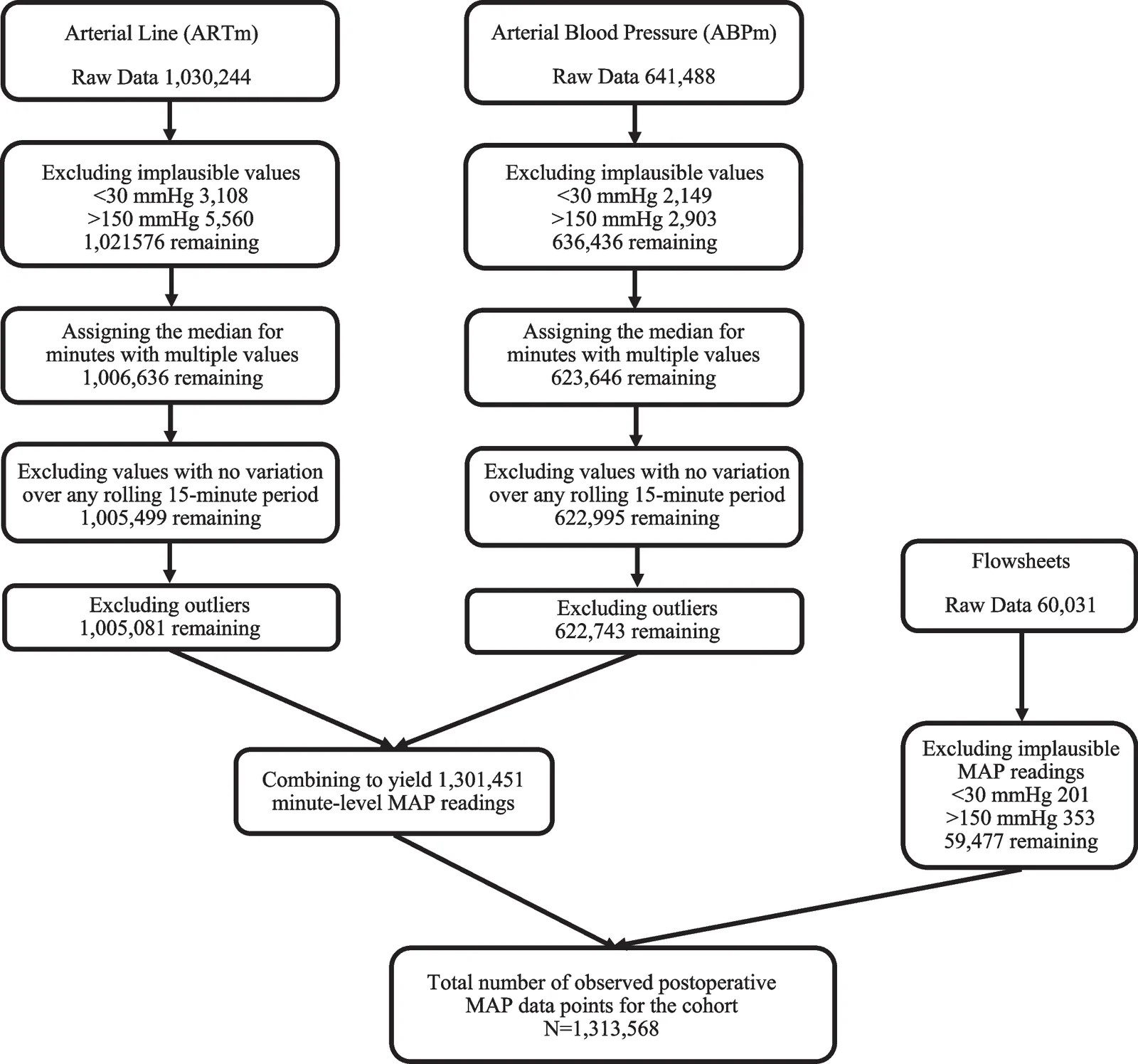
Data Cleaning Flowchart. Flowchart illustrating the multi-step data filtering process for Arterial Line (ARTm), Arterial Blood Pressure (ABPm), and Flowsheets source data. The flowchart tracks the exclusion of implausible values, outliers, and invariant readings, resulting in a total of 1,313,568 min-level postoperative MAP data points observed across the patient cohort

The 1,368 included patients contributed a total of 1,895,133 person-minutes during the first 24 in the ICU. Overall, 1,030,244 raw ARTm and 641,488 raw ABPm values were observed. After removing implausible values (< 30 or > 150 mmHg), assigning medians to minutes with multiple values, excluding non-varying 15-min sequences, and identifying outliers, 1,005,081 ARTm and 622,743 ABPm values were retained. The cleaned datasets were combined to yield 1,301,451 min-level MAP readings. Additionally, nursing flowsheet data populated an additional 12,117 min, bringing total observed postoperative MAP data points to 1,313,568 (Fig. 1).

The remaining 581,565 missing patient-minutes (30.7%) were subsequently resolved using the two-step multiple imputation protocol to generate 50 fully imputed datasets. Using these complete datasets, we calculated the total minutes each patient spent within each of the seven prespecified MAP bands. Finally, individualized longitudinal MAP profiles over the first 24 h in the ICU were constructed as our primary exposure metrics.

To assess the influence of MI on primary exposure metrics, we examined key diagnostic metrics. The between-imputation variance was minimal (< 0.001), while within-imputation variance ranged from < 0.001 to 0.004. Across MAP bands, the relative increase in variance (0.015–0.037) and the fraction of missing information (0.015–0.036) remained low. Relative efficiency values were all near 1.000, indicating that the established longitudinal profiles were highly stable and minimally affected by imputation (Supplemental Digital Content 2).

To validate whether the multiple imputation protocol could reflect real-world physiological variation, we performed a post-hoc sensitivity analysis. First, we isolated a baseline reference cohort of 300 patients with less than 10% missing data during the first 24 h in the ICU. Second, we systematically introduced artificial missingness by randomly deleting observed MAP values totaling 240, 360, 480, 600, and 720 min. Third, these missing segments were re-imputed using the methods we introduced. Framework performance was evaluated by comparing distribution agreement via the percentage of time spent within each of the seven MAP bands. The validation demonstrated strong metric agreement across all five data-loss scenarios (Table 2). Under mild data loss (240 min), absolute distribution deviation across the seven MAP bands ranged from 0.5% to 2.5%. Under extreme data loss (720 min), the deviation remained bounded between 0.8% and 3.2%. However, when incorporating the fully observed data segments, these differences are substantially diluted, bringing the maximum discrepancy across all groups down to a negligible range of 0.5% to 1.5%.

**Table 2:** Distribution Agreement Between True Observed and Imputed Data Within Longitudinal MAP Profiles Across Five Data Missingness Scenarios (N = 300)

MAP Band (mmHg)	True vs Imputed (240 min missing)	True vs Imputed (360 min missing)	True vs Imputed (480 min missing)	True vs Imputed (600 min missing)	True vs Imputed (720 min missing)
< 55	0.70% vs 2.25%	0.75% vs 2.42%	0.75% vs 2.56%	0.77% vs 2.67%	0.75% vs 3.01%
55–59	2.36% vs 3.34%	2.33% vs 3.51%	2.40% vs 3.64%	2.35% vs 3.74%	2.38% vs 3.94%
60–64	8.42% vs 8.94%	8.44% vs 8.93%	8.37% vs 9.21%	8.42% vs 8.85%	8.43% vs 9.20%
65–69	20.26% vs 18.73%	20.44% vs 18.65%	20.44% vs 18.56%	20.36% vs 18.13%	20.44% vs 17.99%
70–74	24.04% vs 21.47%	23.45% vs 21.17%	23.62% vs 20.72%	23.62% vs 20.57%	23.36% vs 20.24%
75–79	17.84% vs 17.00%	17.96% vs 16.80%	17.95% vs 16.66%	17.91% vs 16.73%	18.06% vs 16.09%
≥ 80	26.38% vs 28.26%	26.62% vs 28.51%	26.47% vs 28.65%	26.58% vs 29.31%	26.59% vs 29.53%

## Discussion

This paper describes a framework for leveraging high-frequency postoperative ICU arterial pressure data from EHRs. Through validation of statistical stability and distributional agreement between observed and imputed data, this approach is demonstrated to effectively construct longitudinal profiles that closely align with the actual patterns of postoperative ICU MAP in cardiac surgery patients.

Previous studies characterizing blood pressure exposure metrics have been limited by infrequent sampling or relying on simplified exposure assumptions that overlook continuous physiological fluctuations [1, 3, 6, 7]. To address these limitations, our workflow imputes missing data to generate continuous minute-by-minute MAP profiles, ensuring that every single minute contributes to the finalized exposure metrics. This granular tracking ensures that clinically important transient episodes are better captured rather than mathematically smoothed out. Furthermore, the availability of this minute-by-minute data provides operational flexibility, allowing investigators to calculate diverse exposure summaries and custom blood pressure metrics to meet their specific research objectives.

Our framework also offers several complementary strengths. While currently applied to ICU arterial pressure data following cardiac surgery, it could potentially be extended to other clinical contexts provided that high-frequency blood pressure data are available. Furthermore, wireless, battery-powered vital sign monitoring systems, already available for ward use,[15, 16] might further expand the applications of our method by enabling continuous monitoring for patients who traditionally receive intermittent assessments. However, future work is required to explore and validate these possibilities. Moreover, although archival systems are typically optimized for storage rather than active research, limiting accessibility and interpretability, their value is increasingly recognized. Institutions are investing in systematic collection and preservation of such data, creating opportunities for research and quality improvement [17]. In this study, we extracted raw data from different electronic repositories, including an archival database, to construct our finalized dataset. With comparable effort, similar data architectures at other hospitals could likewise be leveraged, demonstrating that this processing workflow is transferable across different institutions and health record vendor environments. Importantly, our data processing logic does not rely on proprietary hospital hardware or a single EHR vendor, making it highly reproducible across diverse clinical data environments. As institutions continue integrating high-frequency physiological monitoring data into EHR ecosystems, this framework may support larger-scale perioperative research and quality improvement initiatives while maximizing analytical fidelity.

Our study has several limitations. A key limitation is that ICU data missingness may be non-random and informative, as early line removal or early discharge often indicates clinical stability, whereas line dysfunction or transport typically occurs in sicker patients. Although our imputation protocol integrates extensive longitudinal covariates to capture patient acuity, the potential for residual informative missingness remains. Indeed, the underlying complexities of clinically informative missingness may not be fully replicated by our current framework. Additionally, summarizing minute-by-minute MAP records into a cumulative duration within each band may lose temporal sequence information, such as distinguishing between a single sustained hypotensive episode and multiple isolated transient episodes. Furthermore, this approach does not differentiate early from late postoperative hypotension, nor does it account for whether these MAP variations occurred during vasoactive medication use. Tracking these chronological trajectories is clinically important. Therefore, future studies should focus on developing more advanced exposure metrics to fully preserve the temporal structure. Moreover, despite robust cleaning and cross-validation, results remain dependent on source EHR data quality as systemic measurement errors cannot be eliminated.

In summary, we present a reusable workflow for processing high-frequency ICU MAP data. Our method optimizes data extraction, cleaning, and imputation with minimal assumptions. Given its adaptability, generalizability, and scalability, this approach offers a standardized framework to build robust exposure metrics for future clinical research and quality improvement initiatives.

## Supplementary Information

Below is the link to the electronic supplementary material.

Supplementary file1 (DOCX 21 KB)

Supplementary file2 (DOCX 18 KB)

## Data Availability

The clinical data that support the findings of this study are available from Vanderbilt University Medical Center (VUMC). Restrictions apply to the availability of these data, which were used under license for the current study and so are not publicly available. The data are, however, available from the authors upon reasonable request and with the permission of VUMC.
