# Supplementary material for: Leveraging High-Frequency Postoperative ICU Arterial Blood Pressure Data from Electronic Health Records for Research: A Brief Report
Source: J Med Syst. 2026 Oct 14;50(1):147. doi: 10.1007/s10916-026-02472-4 (PMC13668971; doi:10.1007/s10916-026-02472-4)
Supplement: Supplementary file 1 [file 10916_2026_2472_MOESM1_ESM.docx]

| **Supplemental Digital Content 1. Variables Included in the Multiple Imputation Model.** | |
| --- | --- |
| **Category** | **Variable** |
| **Demographics** | Age |
|  | Sex |
|  | Race/ethnicity |
|  | Weight in kg |
|  | Height in cm |
|  | Body mass index (BMI) in kg/m² |
| **Preoperative Clinical Status** | ASA Physical Status classification |
|  | Baseline mean arterial pressure (MAP) |
|  | History of hypertension |
|  | Diabetes mellitus |
|  | Chronic obstructive pulmonary disease (COPD) |
|  | Congestive heart failure (CHF) |
|  | History of neurological disorder |
|  | Peripheral vascular disease |
|  | Liver disease |
|  | Coagulopathy |
|  | Left ventricular ejection fraction |
|  | Last preoperative serum creatinine |
|  | Preoperative estimated glomerular filtration rate (eGFR) |
|  | Preoperative hemoglobin concentration |
|  | Tested positive for COVID-19 within 6 months before surgery |
| **Preoperative Medication Administration** | Acetaminophen (oral, within 6 hours before surgery) |
|  | Gabapentin (oral, within 6 hours before surgery) |
|  | Calcineurin inhibitor administration |
| **Surgical Characteristics** | Date of surgery |
|  | Type of cardiac surgery |
|  | Duration of anesthesia |
|  | Cardiopulmonary bypass duration |
| **Intraoperative Variables** | Intraoperative MAP |
|  | Lowest intraoperative temperature |
|  | Lowest intraoperative hemoglobin concentration |
|  | Intraoperative crystalloid volume |
|  | Intraoperative albumin administration |
|  | Intraoperative red blood cell (RBC) transfusion volume |
|  | Vancomycin administration (pre- or intraoperative) |
|  | Midazolam administration (pre- or intraoperative) |
|  | Dexmedetomidine administration |
|  | Lidocaine administration |
|  | Ketamine administration |
| **Postoperative Variables** | Postoperative MAP |
|  | Postoperative central venous pressure (CVP) |
|  | Postoperative crystalloid volume |
|  | Postoperative albumin administration |
|  | Postoperative RBC transfusion volume |
|  | Acetaminophen administration (oral) |
|  | Gabapentin administration (oral) |
|  | Dexmedetomidine administration |
|  | Lidocaine administration |
|  | Ketamine administration |
|  | Peak norepinephrine dosage |
|  | Peak dobutamine dosage |
|  | Peak epinephrine dosage |
|  | Peak angiotensin II dosage |
|  | Peak vasopressin dosage |
|  | Peak milrinone dosage |
|  | Peak dopamine dosage |
| **Dynamic Hemodynamic Context** | Preceding minute MAP reading |
|  | Subsequent minute MAP reading |
| **Early Postoperative Outcomes** | Acute kidney injury (AKI) within 24 hours to 7 days postoperatively |
|  | Postoperative delirium |
|  | Postoperative stroke |
|  | Unplanned return to operating room for bleeding within 24 hours |
|  | First Sequential Organ Failure Assessment (SOFA) score in ICU |
|  | Lowest cardiac index within 24 hours postoperatively |
| **Hospital Outcomes** | ICU length of stay |
|  | Hospital length of stay |
