# Supplementary material for: Leveraging High-Frequency Postoperative ICU Arterial Blood Pressure Data from Electronic Health Records for Research: A Brief Report
Source: J Med Syst. 2026 Oct 14;50(1):147. doi: 10.1007/s10916-026-02472-4 (PMC13668971; doi:10.1007/s10916-026-02472-4)
Supplement: Supplementary file 2 [file 10916_2026_2472_MOESM2_ESM.docx]

| **Supplemental Digital Content 2. The Validation of Multiple Imputation Results.** | | | | | | |
| --- | --- | --- | --- | --- | --- | --- |
| Postoperative MAP bands | Variance | | | Relative Increase in Variance | Fraction Missing Information | Relative Efficiency |
|  | Between | Within | Total |  |  |  |
| <55 mmHg | 0.0001 | 0.0043 | 0.0044 | 0.0314 | 0.0305 | 0.9994 |
| 55-59 | 0.0001 | 0.0023 | 0.0023 | 0.0261 | 0.0254 | 0.9995 |
| 60-64 | 0.0001 | 0.0021 | 0.0022 | 0.0368 | 0.0355 | 0.9993 |
| 65-69 (reference) | - | - | - | - | - | - |
| 70-74 | 0.0001 | 0.0013 | 0.0014 | 0.0375 | 0.0362 | 0.9993 |
| 75-79 | 0.0000 | 0.0005 | 0.0005 | 0.0151 | 0.0149 | 0.9997 |
| ≥80 mmHg | 0.0000 | 0.0004 | 0.0005 | 0.0365 | 0.0353 | 0.9993 |
